# *CTSC* and Papillon–Lefèvre syndrome: detection of recurrent mutations in Hungarian patients, a review of published variants and database update

**DOI:** 10.1002/mgg3.61

**Published:** 2014-02-11

**Authors:** Nikoletta Nagy, Péter Vályi, Zsanett Csoma, Adrienn Sulák, Kornélia Tripolszki, Katalin Farkas, Ekaterine Paschali, Ferenc Papp, Lola Tóth, Beáta Fábos, Lajos Kemény, Katalin Nagy, Márta Széll

**Affiliations:** 1 Department of Medical Genetics, University of SzegedSzeged, Hungary; 2 Department of Dermatology and Allergology, University of SzegedSzeged, Hungary; 3 Dermatological Research Group of the Hungarian Academy of Sciences, University of SzegedSzeged, Hungary; 4 Department of Periodontology, University of SzegedSzeged, Hungary; 5 Department of Pediatrics, University of SzegedSzeged, Hungary; 6 Mór Kaposi Teaching HospitalKaposvár, Hungary

**Keywords:** Aggressive periodontitis, *CTSC* gene, Haim–Munk syndrome, Papillon–Lefèvre syndrome

## Abstract

Papillon–Lefèvre syndrome (PLS; OMIM 245000) is an autosomal recessive condition characterized by palmoplantar hyperkeratosis and periodontitis. In 1997, the gene locus for PLS was mapped to 11q14-21, and in 1999, variants in the *cathepsin C* gene (*CTSC*) were identified as causing PLS. To date, a total of 75 different disease-causing mutations have been published for the *CTSC* gene. A summary of recurrent mutations identified in Hungarian patients and a review of published mutations is presented in this update. Comparison of clinical features in affected families with the same mutation strongly confirm that identical mutations of the *CTSC* gene can give rise to multiple different phenotypes, making genotype–phenotype correlations difficult. Variable expression of the phenotype associated with the same *CTSC* mutation may reflect the influence of other genetic and/or environmental factors. Most mutations are missense (53%), nonsense (23%), or frameshift (17%); however, in-frame deletions, one splicing variant, and one 5′ untranslated region (UTR) mutation have also been reported. The majority of the mutations are located in exons 5–7, which encodes the heavy chain of the cathepsin C protein, suggesting that tetramerization is important for cathepsin C enzymatic activity. All the data reviewed here have been submitted to the *CTSC* base, a mutation registry for PLS at http://bioinf.uta.fi/CTSCbase/.

## Background

Papillon–Lefèvre syndrome (PLS; OMIM 245000) is a rare form of palmoplantar keratodermas. It was first described by Papillon and Lefèvre (1924). The main characteristic features of PLS are symmetrical palmoplantar hyperkeratosis and periodontal inflammation, causing loss of both the primary and permanent teeth.

Keratoderma in PLS can present in the first 3 months of life, although palmoplantar hyperkeratosis generally first appears in years 1–4 (Haneke 1979). However, several late-onset variants of PLS have also been reported (Bullon et al. 1993; Pilger et al. 2003). Skin symptoms include transgrediens spread with hyperkeratosis of palms and soles. Diffuse hyperkeratosis is the most commonly observed type; however, the punctuate type occurs rarely. Generally, hyperkeratosis in PLS is not severe (Toomes et al. 1999). Psoriasiform lesions may also develop on the elbows, knees, and knuckles (Toomes et al. 1999). As PLS skin lesions are similar to Mal de Meleda (OMIM 248300) lesions, another rare form of palmoplantar keratodermas, PLS was first considered as a variant of Mal de Meleda. Subsequently, it was determined that the two diseases are different forms of palmoplantar keratodermas (Gorlin et al. 1964).

Periodontitis and gingivitis result in the loss of primary and permanent teeth (Gorlin et al. 1964; Toomes et al. 1999; Hart et al. 2000c; Hewitt et al. 2004a,b). As symptoms appear as the teeth erupt, PLS patients typically report two episodes of gingivitis: the first one at ∼3 years of age, leading to the loss of primary teeth (Lundgren and Renvert 2004), the second one at ∼15 years of age, resulting in the loss of permanent teeth (Fardal et al. 1998).

In addition to these symptoms, recurrent skin infections and liver abscesses are frequently reported (de Haar et al. 2004; Pham et al. 2004a,b; Romero-Quintana et al. 2013). Moreover, mild mental retardation, intracranial calcifications, and hyperhidrosis can also occur (Haneke 1979). Japanese patients might have an increased risk of developing melanomas at the sites of hyperkeratosis (Nakajima et al. 2008) than other ethnic groups. The prevalence of the disease is 1–4 cases per million and more than 300 cases have been reported worldwide (Gorlin et al. 1964; Haneke 1979). PLS has been reported to occur in a diverse range of ethnic groups and parental consanguinity has been noted in more than 50% of the cases (Gorlin et al. 1964).

PLS is transmitted as an autosomal recessive condition affecting males and females equally. PLS was independently mapped to chromosome 11q14-21 by three groups (Fischer et al. 1997; Laass et al. 1997; Hart et al. 1998). In the mapped region, the causative *cathepsin C* gene (*CTSC*) was independently identified by two groups (Hart et al. 1999; Toomes et al. 1999). The *CTSC*, GenBank accession number NM_001814.4 spans over 46 kb and contains seven exons and six introns (Toomes et al. 1999). According to the Ensemble genome browser (http://www.ensembl.org), this gene has nine splice variants. Of these, five occur in protein coding regions; the remaining four are noncoding transcripts.

*CTSC* encodes the cathepsin C protein (dipeptidyl-peptidase I), a lysosomal exo-cysteine proteinase belonging to the peptidase C1 family. Cathepsin C is an oligomeric enzyme composed of four identical subunits (Dolenc et al. 1995; Paris et al. 1995). Each subunit contains three different polypeptides – heavy, light, and propeptide chains – which are held together by noncovalent interactions (Cigić et al. 1998). The C-terminus of the propeptide is cleaved upon activation. The residual propeptide is cleaved into two peptides, which are held together by a disulfide bond (Cigić et al. 1998).

Cathepsin C has the ability to remove dipeptides from the amino terminus of proteins and is involved in the zymogen activation of serine proteases. This activity was proposed to play a role in epithelial differentiation and desquamation (Toomes et al. 1999).

In 1999, the first eight mutations of the *CTSC* gene were identified in consanguineous PLS families (Toomes et al. 1999). Since 1999, several reports have described mutations in the *CTSC* gene in different PLS cases from around the world (Table 1). *CTSC* mutations have also been reported in patients with Haim–Munk syndrome (HMS, OMIM 245010), also characterized by palmoplantar hyperkeratosis and periodontal inflammation, as well as arachnodactly, acroosteolysis, pesplanus, and onychogryposis (Hart et al. 2000b). *CTSC* mutations were also found in aggressive periodontitis (AP1, OMIM 170650), which is characterized by severe periodontal inflammation leading to tooth loss without the presence of skin symptoms (Hart et al. 2000c; Hewitt et al. 2004a,b).

**Table 1:** Summary of studies reporting *CTSC* gene mutations

Location	Mutation	Ethnicity	References
5′UTR	c.-55C>A	Slovenian	Kosem et al. (2012)
Exon 1	c.21delG, c.72C>A, c.90C>A, c.96T>G, c.113delCCTG, c.116G>C, c.145C>T	Chinese, French, Indian, Mexican, Moroccan, North African, North American, Puerto-Rican, Thai	Lefèvre et al. (2001), Nakano et al. (2001), Zhang et al. (2002), Allende et al. (2003), Selvaraju et al. (2003), Hewitt et al. (2004a,b), Pham et al. (2004a,b), Nitta et al. (2005), Yang et al. (2007), Kurban et al. (2010)
Exon 2	c.199del24, c.203T>G, c.205C>T, c.267delGG	Brazilian, Chinese, Indian, Mexican	Hart et al. (2000a,b,c), Selvaraju et al. (2003), Pallos et al. (2010), Romero-Quintana et al. (2013)
Exon 3	c.322A>T, c.380A>C, c.386T>A, c.415G>A, c.436delT, c.444insATGT, c.458C>T	Chinese, Egyptian, French, German, Indian, North American, Scotish, Turkish	Hart et al. (2000a,b,c), Lefèvre et al. (2001), Hewitt et al. (2004a,b), Cagli et al. (2005), Yang et al. (2007), Noack et al. (2008a,b), Kobayashi et al. (2013)
Intron 3	c.485-1G>A	Egyptian, Jordanian	Toomes et al. (1999)
Exon 4	c.555G>A, c.566delCATACAT, c.587T>C, c.622insC, c.628C>T, c.629delGA	Algerian, Brazilian, German, Hungarian, Indian, Iranian, Lebanese, Moroccan, Russian, Turkish,	Hart et al. (2000a,b,c, 2002), Cury et al. (2002), Noack et al. (2004, 2008a,b), Cury et al. (2005), Wani et al. (2006), Farkas et al. (2013)
Exon 5	c.704G>A, c.706G>T, c.711del14, c.739A>C, c.745G>T, c.748C>T, c.755A>T, c.756ins130	Algerian, Chinese, Egyptian, Eritrean, Indian, Iranian, North American, Pakistanian, Spanish, Turkish	Toomes et al. (1999), Hart et al. (2000a,b,c), Allende et al. (2001), Lefèvre et al. (2001), Hewitt et al. (2004a,b), Jouary et al. (2008), Wen et al. (2012)
Exon 6	c.778T>C, c.815G>A, c.815G>C, c.851G>A, c.854C>T, c.856C>T, c.857A>G, c.872G>A, c.880T>C	Belgian, Chinese, French, Holland, Indian, Lebanese, Moroccan, North American, Russian, Saudi, Spanish, Sri Lankan, Turkish	Hart et al. (1999, 2000a,b,c), Toomes et al. (1999), Allende et al. (2001, 2003), Lefèvre et al. (2001), Zhang et al. (2002), de Haar et al. (2004), Hewitt et al. (2004a,b), Pham et al. (2004a,b), Yang et al. (2007), Noack et al. (2008a,b)
Exon 7	c.890G>T, c.898G>A, c.899G>A, c.901G>A, c.901G>T, c.902G>T, c.910T>A, c.912C>A, c.923G>A, c.935A>G, c.947T>G, c.956A>G, c.984delTTCTCCA, c.1015C>T, c.1019A>G, c.1028delCT, c.1040A>G, c.1047delA, c.1056delT, c.1131T>G, c.1141delC, c.1156G>C	Egyptian, French, German, Indian, Indian–Pakistanian, Iranian, Japanese, Jordanian, Martinique, North American, Panamanian, Saudi, Sri Lankan, Turkish, Vietnamese	Hart et al. (1999, 2000a,b,c), Toomes et al. (1999), Lefèvre et al. (2001), Nakano et al. (2001), Zhang et al. (2001), Selvaraju et al. (2003), de Haar et al. (2004, 2005), Hewitt et al. (2004a,b), Noack et al. (2004, 2008a,b), Wani et al. (2006), Jouary et al. (2008), Castori et al. (2009), Wen et al. (2012)

To date, a total of 75 mutations have been reported for the *CTSC* gene. The majority of the mutations (97%) were reported in PLS cases, while only a few mutations (3%) were reported in HMS or AP1 cases. Note that some mutations were detected in two different disease entities: c.1040A>G p.Tyr347Cys was reported for AP1 and also for classic PLS families (Toomes et al. 1999; Hart et al. 2000c; Hewitt et al. 2004a,b), c.145C>T p.Gln49X was reported for HMS and for PLS pedigrees (Selvaraju et al. 2003; Rai et al. 2010) and c.857A>G p.Gln286Arg was present in patients either with the HMS or with the PLS phenotype (Hart et al. 2000b). Therefore, PLS, HMS, and AP1 are not different entities; they represent the phenotypic spectrum of a single disease.

## Database

A PubMed (http://www.ncbi.nlm.nih.gov/pubmed) literature search was performed to identify all known *CTSC* mutations. In addition, Hungarian pedigrees with PLS were screened for *CTSC* mutations and added to this article. All available information about mutation carriers have been uploaded to the CTSCbase, a mutation registry for PLS (Piirilä et al. 2006). This database is included in the Human Genome Variation Society (HGVS) (http://www.HGVS.org) list of locus-specific databases. The database can be visited at http://bioinf.uta.fi/CTSCbase/ and has been updated with data from the literature as well as unpublished variants identified in Hungarian PLS pedigrees.

## Summary of Clinical Findings for Hungarian PLS Patients With Recurrent Mutations

In Hungary, mutation screening for the *CTSC* gene has been available since 2011. Screening is performed with direct sequencing of all coding regions and flanking introns of the *CTSC* gene. Once a putative causative variant was identified in a patient, the available, clinically symptom-free family members and unrelated, healthy control individuals were also investigated.

We have recently identified a Hungarian family with two sisters affected with mild palmoplantar hyperkeratosis and severe periodontitis leading to the loss of all primary teeth. These patients carried the recurrent c.566delCATACAT p.Thr189fsX199 frameshift mutation in a homozygous form (Farkas et al. 2013). An unaffected sister and the parents carried the same mutation in a heterozygous form. The family was not aware of consanguinity. This frameshift mutation has also been previously published for two Moroccan PLS patients presenting variation in the severity of the skin symptoms (Noack et al. 2008a,b).

In another Hungarian family with two sisters presenting severe tooth loss and different degrees of palmoplantar hyperkeratosis (severe and mild), the sisters were found to carry the c.901G>Ap.Gly301Ser missense mutation in a homozygous form (data not published). The family was not aware of consanguinity. This mutation has also been previously published for a German patient with typical PLS skin symptoms (Noack et al. 2008a,b).

In a pair of unrelated Hungarian patients with typical PLS phenotype (a 25-year-old male patient and a 39-year-old female patient), we have identified the c.748C>Tp.Arg250X homozygous nonsense mutation (data not published). Unfortunately, both of these patients were reared in state care and have no known relatives; therefore, investigation of the family was not possible. The fact that both individuals carry the same mutation raises the possibility that these patients are relatives. This mutation has also been previously published in the literature in a Turkish PLS family (Hart et al. 2000a).

## Variants in the *CTSC* Gene

**Figure 1:**
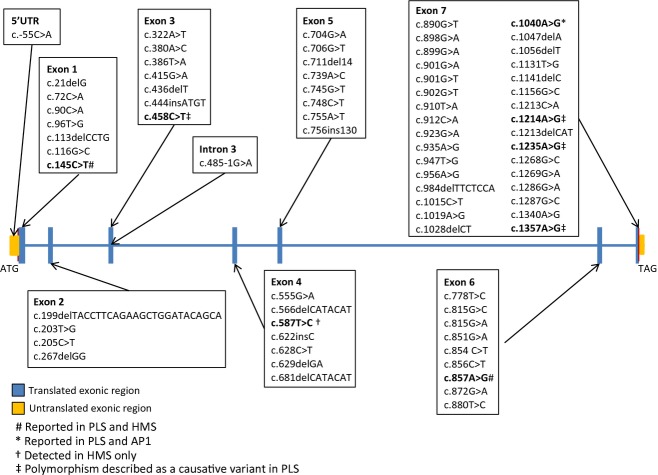
Schematic drawing of the *CTSC* gene, indicating the positions of mutations leading to PLS, HMS, and AP1. Identical mutations can lead to different diseases. The involvement of mutations in specific diseases is indicated as follows: #, PLS and HMS; *, PLS and AP1; †, HMS; ‡, common missense variants reported as causative for PLS.

To date, a total of 75 mutations have been identified for the *CTSC* gene, all of which are registered in the CTSCbase. Mutations are named according to HGVS nomenclature guidelines (http://www.HGVS.org) and numbered with respect to the *CTSC* gene reference sequence (ENSG00000109861 corresponding to the *CTSC* gene transcript ENST00000227266). The 75 unique mutations – point mutations, small deletions, and insertions – are summarized in Figure 1.

Of the reported 75 mutations, 53% are missense (*n* = 40), 23% are nonsense (*n* = 17) and 17% are frameshift (*n* = 13) variants. There are two in-frame deletions, one intronic splice-site variant and one point mutation in the 5′ untranslated region (UTR) of the *CTSC* gene. The majority (75%, *n* = 56) of the mutations has only been reported once. Among these, 65% (*n* = 36) were present in homozygous form in the investigated patients, while 35% (*n* = 20) occurred in a compound heterozygous form. Recurrent mutations (25% of all mutations, *n* = 19) occurred both in homozygous and in compound heterozygous forms and were detected in geographically distant, unrelated families, suggesting mutational clustering on the *CTSC* gene. However, there are reports suggesting that an initial founder effect and subsequent migration of carriers can lead to the presence of the same mutation in geographically distant and unrelated families (Zhang et al. 2001; Kurban et al. 2009).

Known mutations that have been sequenced are unequally distributed on the *CTSC* gene. Half of the mutations (53%, *n* = 41) are located within exons 5–7, encoding amino acids 231–394 in the heavy-chain region. Of the remaining half, 16% (*n* = 12) are located within exons 1–3 encoding amino acids 25–134 in the exclusion domain, 12% (*n* = 9) are located within the second half of exon 7 encoding amino acids 395–463 in the light-chain region, 13% (*n* = 10) are located within exon 4 and the first half of exon 5 encoding amino acids 135–230 in the propeptide region, 3% (*n* = 2) are located in the 5′ end of exon 1 encoding amino acids 1–24 in the signal peptide region and 3% (*n* = 2) are located within UTRs. Note, not all mutations have been identified by DNA sequencing.

## Homozygous Mutations

**Figure 2:**
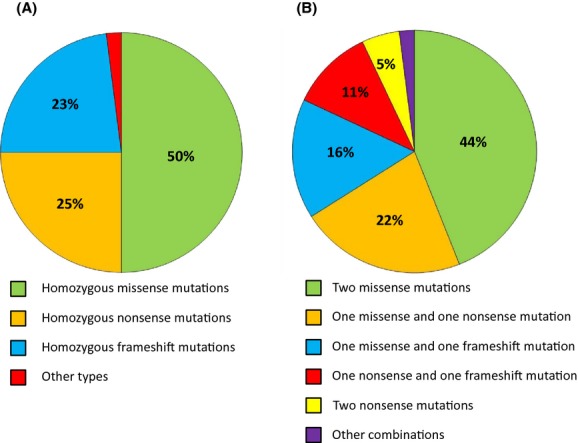
The frequency of mutation types reported for PLS patients in (A) homozygous and (B) compound heterozygous forms.

To date, 68% of all identified *CTSC* mutations (*n* = 75) were reported in a homozygous form in PLS patients. Of these mutations, 85% (*n* = 64) were present only in homozygous form in PLS patients, while 15% (*n* = 11) were also detected in a compound heterozygous state. Among the homozygous mutations, 50% (*n* = 32) were missense, 25% (*n* = 16) nonsense, 23% (*n* = 15) frameshift mutations, and 2% (*n* = 1) were other types of mutations (Fig. 2A).

## Missense Variants

**Figure 3:**
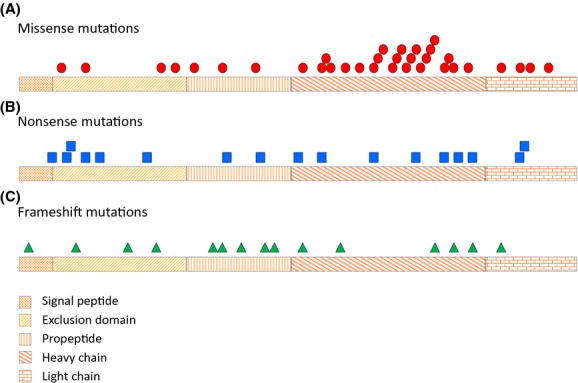
Distribution of mutations on the cathepsin C protein: (A) missense, (B) nonsense, and (C) frameshift.

Missense mutations account for approximately half (53%, *n* = 41) of all *CTSC* gene mutations identified to date. Missense mutations occur in all coding regions of the gene; however, the majority occurs in exons 5–7, encoding the heavy-chain region of the cathepsin C protein (Fig. 3A), which is thought to be important for enzyme activity (Turk et al. 2001).

In addition to mutations of the *CTSC* gene, it is important to note that some polymorphisms are common for this gene. For example, the c.458C>T p.Thr153Ile missense variant, which corresponds to variant rs217086, occurs at a residue that is conserved in mammals and is located in the portion of the propeptide that is cleaved upon activation (Hart et al. 2000a). The c.458C>T p.Thr153Ile polymorphism has been indentified in several PLS families, but does not have a causative role in the development of PLS (Allende et al. 2001; Nakano et al. 2001; de Haar et al. 2004; Romero-Quintana et al. 2013).

Further missense variants of the *CTSC* gene reported in PLS families have also been detected as rare polymorphisms as well: c.1214A>Gp.His405Arg corresponds the rs151269219 polymorphism (de Haar et al. 2005; Noack et al. 2008a,b), c.1235A>Gp.Tyr412Cys to the rs28937571 (Hewitt et al. 2004a,b), and c.1357A>Gp.Ile453Val to the rs3888798 polymorphism (Nakano et al. 2001). All of these missense polymorphisms affect the light-chain region of the cathepsin C protein, which is important in the tetramerization of the matured cathepsin C protein. Their eventual pathogenic role should be confirmed or excluded by further studies. It is also possible that these polymorphisms share a common haplotype and are markers of other underlying, still uncharacterized, genetic abnormalities in these PLS patients.

## Nonsense Variants

Nonsense mutations account for 23% (*n* = 17) of the pathogenic mutations identified for the *CTSC* gene to date. Nonsense mutations occur in all coding regions of the gene; however, the majority is located in exons 5–7, encoding the heavy-chain region of the cathepsin C protein (Fig. 3B), which is thought to be important for enzyme activity (Turk et al. 2001).

## Frameshift Variants

After missense and nonsense mutations, frameshift mutations of the *CTSC* gene are the most common, accounting for 17% (*n* = 13) of the mutations identified to date. Frameshift mutations occur in all coding regions of the gene; however, the majority is located in exons 4–5 encoding the propeptide region of the cathepsin C protein (Fig. 3C). These mutations might influence the cleavage and the activation processes of the precursor cathepsin C (Turk et al. 2001).

## Other Deletions

Two in-frame deletions have been reported in PLS patients. The c.199delTACCTTCAGAAGCTGGATACAGCA deletion corresponding to p.Tyr67_Tyr75del was detected in compound heterozygous form in combination with the c.458C>T missense variant corresponding to p.Thr153Ile (Hart et al. 2000a). This missense mutation is a common polymorphism with no pathogenic role, as determined in subsequent studies (Allende et al. 2001; Nakano et al. 2001; de Haar et al. 2004; Romero-Quintana et al. 2013). The c.1213delCAT p.His405del in-frame deletion was reported in homozygous form in an Indian PLS patient (Wani et al. 2006). A large intragenic deletion of exons 3–7 was observed for another PLS patient in compound heterozygous form, in combination with another missense mutation, c.1156G>C p.Gly386Arg (Jouary et al. 2008).

## Splicing Variant

To date, only one pathogenic splice-site mutation has been reported for the *CTSC* gene (Toomes et al. 1999). This single-nucleotide change occurs at the splice-acceptor site (5′ end of exon 3) c.485-1G>A (c.IVS3-1G>A).

## UTR Variant

Only one pathogenic mutation has been identified in an UTR of the *CTSC* gene: a single-nucleotide change c.-55C>A at the 5′ end (Kosem et al. 2012). The mutation results in complete loss of *CTSC* mRNA expression and cathepsin C activity (Kosem et al. 2012). In silico analysis suggested that the mutation disrupts the binding sites for AP-2 and Sp transcription factors.

## Compound Heterozygous Mutations

To date, 32% (*n* = 23) of all identified *CTSC* mutations (*n* = 75) were detected in a compound heterozygous form. The most frequent (44%, *n* = 10) compound heterozygotes involved two heterozygous missense mutations. The combination of a heterozygous missense and a heterozygous nonsense mutation occurred in 22% (*n* = 5) of the cases, a heterozygous missense and a heterozygous frameshift mutation in 16% (*n* = 4), a heterozygous nonsense and a heterozygous frameshift mutation in 11% (*n* = 3), and two heterozygous nonsense mutations in 5% (*n* = 1) (Fig. 2B).

## Ethnic Variation

PLS has been reported in a diverse range of ethnic groups from all over the world. A quarter (25%, *n* = 19) of the mutations have been reported twice or more in different ethnic groups. One of the most frequently reported missense mutation, the c.815G>Cp.Arg272Pro variant, has been detected in Lebanese, Turkish, Saudi, Holland, Russian and French PLS patients (Toomes et al. 1999; Lefèvre et al. 2001; Zhang et al. 2002; de Haar et al. 2004; Pham et al. 2004a,b; Noack et al. 2008a,b), while another frequent nonsense mutation, c.96T>Gp.Tyr32X, has been observed in PLS patients from Mexico and France (Lefèvre et al. 2001; Zhang et al. 2002; Pham et al. 2004a,b). Moreover, a common frameshift mutation, c.566delCATACAT p.Thr189fsX200, has been found in Hungarian and Moroccan PLS patients (Noack et al. 2008a,b; Farkas et al. 2013).

Haplotype analyses of different PLS cases carrying identical mutations revealed that these relatively frequent mutations resulted from independent founder events. Two Turkish families carrying the same homozygous nonsense mutation (c.856C>T p.Gln286X exhibited different haplotypes, suggesting that the same mutation arose in the two families independently (Hart et al. 1998, 2000a).

## Biological Relevance

Cathepsin C is a lysosomal cysteine protease that was first characterized as an activator of serine proteases from immune and inflammatory cells (Turk et al. 2001). Cell lines derived from cathepsin C-deficient mice fail to activate groups of serine proteases. Unprocessed proteases zymogens included granzymes A, B, and C, cathepsin G, neutrophil elastase, and chymase (Adkison et al. 2002).

The encoded cathepsin C precursor contains 463 amino acids and includes a signal peptide (24 amino acids), an exclusion domain (110 amino acids), a propeptide (96 amino acids), as well as heavy-(164 amino acids) and light-(69 amino acids) chain regions (Turk et al. 2001; Hewitt et al. 2004a,b). Precursor cathepsin C is processed into the mature form by at least four cleavages of the polypeptide (Turk et al. 2001; Adkison et al. 2002). The signal peptide is removed during translocation or secretion of the protein (Turk et al. 2001; Adkison et al. 2002). The exclusion domain is retained in the mature enzyme and separated from the heavy and light chains by excision of a minor C-terminal portion of the propeptide region. The heavy and light chains are also generated by cleavage (Turk et al. 2001; Adkison et al. 2002).

**Figure 4:**
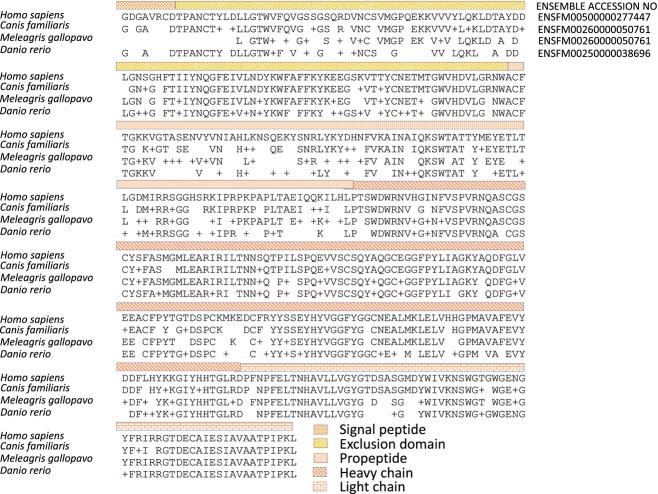
Conservation of cathepsin C protein sequence in vertebrates.

According to a BLAST (http://blast.ncbi.nlm.nih.gov/) search, the cathepsin C protein is highly conserved in vertebrates: the human cathepsin C shows 82% sequence similarity with the sequence from dog, 70% with turkey, and 63% with frog and zebrafish (Fig. 4). The most highly conserved regions are the heavy chain, the light chain, and the C-terminal portion of the exclusion domain, which is thought to be important for enzyme activity.

Half (53%, *n* = 40) of all *CTSC* gene mutations affect the heavy-chain domain and result in different positioning of its N-terminus. As the N-terminal region is involved in oligomer contacts with the N-terminal region of the light chain, the mutation may interfere with tetramer formation (Turk et al. 2001). This finding indicates that tetramerization of the cathepsin C enzyme is crucial for its function. The majority of the two most common types of *CTSC* mutations (missense and nonsense) affect this domain (Fig. 3A and B).

Sixteen percent (*n* = 12) of all *CTSC* mutations affect the exclusion domain, which blocks access to the active site and prevents substrates from binding any part except their N-termini. Thirteen mutations were detected in the exclusion domain; of these, six are nonsense variants, four are missense mutations, and three are deletions (two resulting in frameshift and one in an in-frame deletion).

Thirteen percent (*n* = 10) of all *CTSC* gene mutations affect the propeptide fragment, which plays a pivotal role in the activation of the cathepsin C precursor. The majority of frameshift mutations are located in this domain (Fig. 3C).

Twelve percent (*n* = 9) of all mutations affect the light-chain domain, which is important for tetramerization of the mature enzyme: four are missense mutations, two are nonsense variants and one is an in-frame deletion. Three common missense variants, rs151269219, rs28937571, and rs3888798 are also located in this domain (Nakano et al. 2001; Hewitt et al. 2004a,b; de Haar et al. 2005; Noack et al. 2008a,b).

Three percent (*n* = 3) of all mutations are located in the signal peptide region, presumably affecting the translocation or secretion of the protein: one nonsense mutation and one frameshift variant (Lefèvre et al. 2001; Hewitt et al. 2004a,b; Kurban et al. 2010).

## Clinical and Diagnostic Relevance

Historically, PLS was initially considered a variant of Mal de Meleda, due to the similarity of the skin lesions. Subsequently, the two diseases were determined to be different forms of palmoplantar keratodermas (Gorlin et al. 1964). In addition to palmoplantar hyperkeratosis, periodontal inflammation is a main feature of PLS. Clinical diagnosis of HMS, an allelic variant of PLS, is based on the presence of arachnodactly, acroosteolysis, pesplanus, and onychogryposis in addition to palmoplantar hyperkeratosis and periodontal inflammation (Hart et al. 2000b). AP1, which can be also considered a variable expression of the PLS phenotype, is characterized by periodontal inflammation and the lack of other symptoms. All the three entities develop as a consequence of *CTSC* mutations. Identification of a *CTSC* mutation gives a definite diagnosis of PLS, HMS, or AP1 depending on the presented clinical symptoms. In contrast, the absence of *CTSC* mutation suggests a diagnosis of another palmoplantar keratoderma or nonsyndromic tooth abnormality.

Analysis of data reported for Hungarian PLS patients revealed 75 *CTSC* gene mutations. The most frequent mutations are recurrent and are reported both as homozygous and as compound heterozygous. The identification of the most frequent *CTSC* mutations has great clinical significance, as they highlight regions of the gene that are important for the development of the disease. The most frequent mutations of the *CTSC* gene and their most common associations are summarized in Table 2. Approximately half 53% (*n* = 40) of the all 75 mutations are located within exons 5–7, encoding the heavy-chain region of the cathepsin C protein. Three types mutations accounted for 93% (*n* = 61) of *CTSC* gene mutations: missense 53% (*n* = 41), nonsense 23% (*n* = 17), and frameshift 17% (*n* = 13). In addition, the majority of missense, nonsense, and frameshift mutations occur in exons 5–7.

**Table 2:** The most frequent compound heterozygous pathogenic combinations of *CTSC* mutations

Mutation on Allele 1	Mutation type	Mutation on Allele 2	Mutation type	References
c.96T>G p.Tyr32X	Nonsense	c.380A>C p.His127Pro	Missense	Lefèvre et al. (2001), Zhang et al. (2002), Pham et al. (2004a,b)
		c.815G>A p.Arg272His	Missense	
c.322A>T p.Lys108X	Nonsense	c.436delT p.Ser146fsX30	Frameshift	Noack et al. 2008a,b
		c.504C>G p.Tyr168X	Nonsense	
c.415G>A p.Gly139Arg	Missense	c.72C>A p.Cys24X	Nonsense	Hewitt et al. (2004a,b), Cagli et al. (2005), Yang et al. (2007)
		c.706G>T p.Asp236Tyr	Missense	
		c.778T>C p.Ser260Pro	Missense	
		c.1141delC p.Leu381fsX13	Frameshift	
c.706G>T p.Asp236Tyr	Missense	c.415G>A p.Gly139Arg	Missense	Allende et al. (2001), Hewitt et al. (2004a,b)
		c.872G>A p.Cys291Tyr	Missense	
c.815G>C p.Arg272Pro	Missense	c.96T>G p.Tyr32X	Nonsense	Toomes et al. (1999), Lefèvre et al. (2001), Zhang et al. (2002), de Haar et al. (2004), Pham et al. (2004a,b), Noack et al. (2008a,b)
		c.1141delC p.Leu381fsX13	Frameshift	
c.1141delC p.Leu381fsX13	Frameshift	c.415G>A p.Gly139Arg	Missense	Lefèvre et al. 2001
		c.815G>C p.Arg272Pro	Missense	

## Genotype–Phenotype Correlations

In general, no strict genotype–phenotype correlations have been identified for PLS. Analysis of *CTSC* mutation location (i.e., within or outside the coding regions) suggested that mutations located outside coding regions are more likely to be associated with transgression of the lesions (Hart et al. 2000a), although this hypothesis has not been confirmed (Selvaraju et al. 2003; de Haar et al. 2004; Hewitt et al. 2004a,b). It was also suggested that *CTSC* gene mutations with little functional consequences are putative causes of more common types of early-onset periodontal disease (Hart et al. 2000c), but this observation has also not been confirmed (Hewitt et al. 2004a,b).

Mutations in the *CTSC* gene can lead to the development of HMS or AP1 as well as PLS. The common characteristic of these three entities is periodontal inflammation (Hart et al. 2000b; Hewitt et al. 2004a,b; Cury et al. 2005). While all three diseases involve tooth abnormalities, PLS and HMS also involve characteristic skin symptoms of palmoplantar hyperkeratosis (Hart et al. 2000b; Hewitt et al. 2004a,b; Cury et al. 2005). HMS is further characterized by arachnodactly, acroosteolysis, pesplanus, and onychogryphosis (Hart et al. 2000b; Hewitt et al. 2004a,b; Cury et al. 2005).

Several reports indicate that identical mutations of the *CTSC* gene can give rise to multiple different phenotypes: the c.1040A>G p.Tyr347Cys missense mutation can lead either PLS or AP1 (Toomes et al. 1999; Hart et al. 2000c; Hewitt et al. 2004a,b) and the c.145C>T p.Gln49X nonsense mutation results either in HMS or PLS (Selvaraju et al. 2003; Rai et al. 2010). Hart et al. (2000b) reported that the c.857A>G p.Gln286Arg missense mutation can also contribute to the development of HMS and PLS (Hart et al. 2000b) (Fig. 1). Variable expression of the phenotype associated with the *CTSC* mutation may reflect the influence of other genetic and/or environmental factors (Hart et al. 2000a).

## Future Prospects

To date, the comparison of *CTSC* gene mutations has not yet resulted in the identification of genotype–phenotype correlations. Future efforts might provide insight into these correlations and elucidate the mechanism of the different phenotypic variants – PLS, HMS, and AP1 – of the disease. We believe that, to improve molecular analysis of the *CTSC* gene, it is necessary to promote both better awareness of the PLS, HMS, and AP1 phenotypic variants of the same disease and better understanding of the underlying molecular mechanisms. The availability of the extended clinical findings from *CTSC* mutation carriers, as provided by the CTSCbase, is critical for furthering both our understanding of the disease and the development of causative therapies that will be more specific and effective than the symptomatic treatments currently available for patients with PLS, HMS, and AP1 variants.

## References

[b1] Adkison AM, Raptis SZ, Kelley DG, Pham CT (2002). Dipeptidyl peptidase I activates neutrophil-derived serine proteases and regulates the development of acute experimental arthritis. J. Clin. Invest.

[b2] Allende LM, García-Pérez MA, Moreno A, Corell A, Carasol M, Martínez-Canut P (2001). Cathepsin C gene: first compound heterozygous patient with Papillon-Lefevre syndrome and a novel symptomless mutation. Hum. Mutat.

[b3] Allende LM, Moreno A, de Unamuno P (2003). A genetic study of cathepsin C gene in two families with Papillon-Lefevre syndrome. Mol. Genet. Metab.

[b4] Bullon P, Pascual A, Fernandez-Novoa MC, Borobio MV, Muniain MA, Camacho F (1993). Late onset Papillon-Lefèvre syndrome? A chromosomic, neutrophil function and microbiological study. J. Clin. Periodontol.

[b5] Cagli NA, Hakki SS, Dursun R, Toy H, Gokalp A, Ryu OH (2005). Clinical, genetic, and biochemical findings in two siblings with Papillon-Lefevre syndrome. J. Periodontol.

[b6] Castori M, Madonna S, Giannetti L, Floriddia G, Milioto M, Amato S (2009). Novel CTSC mutations in a patient with Papillon-Lefèvre syndrome with recurrent pyoderma and minimal oral and palmoplantar involvement. Br. J. Dermatol.

[b7] Cigić B, Krizaj I, Kralj B, Turk V, Pain RH (1998). Stoichiometry and heterogeneity of the pro-region chain in tetrameric human cathepsin C. Biochim. Biophys. Acta.

[b8] Cury VF, Costa JE, Gomez RS, Boson WL, Loures CG, De ML (2002). A novel mutation of the cathepsin C gene in Papillon-Lefevre syndrome. J. Periodontol.

[b9] Cury VF, Gomez RS, Costa JE, Friedman E, Boson W, De Marco L (2005). A homozygous cathepsin C mutation associated with Haim-Munk syndrome. Br. J. Dermatol.

[b10] Dolenc I, Turk B, Pungercic G, Ritonja A, Turk V (1995). Oligomeric structure and substrate induced inhibition of human cathepsin C. J. Biol. Chem.

[b11] Fardal O, Drangsholt E, Olsen I (1998). Palmar plantar keratosis and unusual periodontal findings: observations from a family of 4 members. J. Clin. Periodontol.

[b12] Farkas K, Paschali E, Papp F, Vályi P, Széll M, Kemény L (2013). A novel seven-base deletion of the CTSC gene identified in a Hungarian family with Papillon-Lefèvre syndrome. Arch. Dermatol. Res.

[b13] Fischer J, Blanchet-Bardon C, Prud'homme JF, Pavek S, Steijlen PM, Dubertret L (1997). Mapping of Papillon-Lefevre syndrome to the chromosome 11q14 region. Eur. J. Hum. Genet.

[b14] Gorlin RJ, Sedano H, Anderson VE (1964). The syndrome of palmar-plantar hyperkeratosis and premature periodontal destruction of the teeth: a clinical and genetic analysis of the Papillon-Lefevre syndrome. J. Pediatr.

[b15] de Haar SF, Jansen DC, Schoenmaker T, De Vree H, Everts V, Beertsen W (2004). Loss-of-function mutations in cathepsin C in two families with Papillon-Lefevre syndrome are associated with deficiency of serine proteinases in PMNs. Hum. Mutat.

[b16] de Haar SF, Mir M, Nguyen M, Kazemi B, Ramezani GH, Everts V (2005). Gene symbol: CTSC. Disease: Papillon-Lefevre syndrome. Hum. Genet.

[b17] Haneke E (1979). The Papillon-Lefevre syndrome: keratosis palmoplantaris with periodontopathy: report of a case and review of the cases in the literature. Hum. Genet.

[b18] Hart TC, Bowden DW, Ghaffar KA, Wang W, Cutler CW, Cebeci I (1998). Sublocalization of the Papillon-Lefevre syndrome locus on 11q14-q21. Am. J. Med. Genet.

[b19] Hart TC, Hart PS, Bowden DW, Michalec MD, Callison SA, Walker SJ (1999). Mutations of the cathepsin C gene are responsible for Papillon-Lefevre syndrome. J. Med. Genet.

[b20] Hart PS, Zhang Y, Firatli E, Uygur C, Lotfazar M, Michalec MD (2000a). Identification of cathepsin C mutations in ethnically diverse Papillon-Lefevre syndrome patients. J. Med. Genet.

[b21] Hart TC, Hart PS, Michalec MD, Zhang Y, Firatli E, Van Dyke TE (2000b). Haim-Munk syndrome and Papillon-Lefevre syndrome are allelic mutations in cathepsin C. J. Med. Genet.

[b22] Hart TC, Hart PS, Michalec MD, Zhang Y, Marazita ML, Cooper M (2000c). Localisation of a gene for prepubertal periodontitis to chromosome 11q14 and identification of a cathepsin C gene mutation. J. Med. Genet.

[b23] Hart PS, Pallos D, Zhang Y, Sanchez J, Kavamura I, Brunoni D (2002). Identification of a novel cathepsin C mutation (p. W185X) in a Brazilian kindred with Papillon-Lefevre syndrome. Mol. Genet. Metab.

[b24] Hewitt C, McCormick D, Linden G, Turk D, Stern I, Wallace I (2004a). The role of cathepsin C in Papillon-Lefevre syndrome, prepubertal periodontitis, and aggressive periodontitis. Hum. Mutat.

[b25] Hewitt C, Wu CL, Hattab FN, Amin W, Ghaffar KA, Toomes C (2004b). Coinheritance of two rare genodermatoses (Papillon-Lefevre syndrome and oculocutaneous albinism type 1) in two families: a genetic study. Br. J. Dermatol.

[b26] Jouary T, Goizet C, Coupry I, Redonnet-Vernhet I, Levade T, Burgelin I (2008). Detection of an intragenic deletion expands the spectrum of CTSC mutations in Papillon-Lefèvre syndrome. J. Invest. Dermatol.

[b27] Kobayashi T, Sugiura K, Takeichi T, Akiyama M (2013). The novel CTSC homozygous nonsense mutation p.Lys106X in a patient with Papillon-Lefèvre syndrome with all permanent teeth remaining at over 40 years of age. Br. J. Dermatol.

[b28] Kosem R, Debeljak M, RepičLampret B, Kansky A, Battelino T, TrebušakPodkrajšek K (2012). Cathepsin C gene 5′-untranslated region mutation in Papillon-Lefèvre syndrome. Dermatology.

[b29] Kurban M, Wajid M, Shimomura Y, Bahhady R, Kibbi AG, Christiano AM (2009). Evidence for a founder mutation in the cathepsin C gene in three families with Papillon-Lefèvre syndrome. Dermatology.

[b30] Kurban M, Cheng T, Wajid M, Kiuru M, Shimomura Y, Christiano AM (2010). A novel mutation in the cathepsin C gene in a Pakistani family with Papillon-Lefevre syndrome. J. Eur. Acad. Dermatol. Venereol.

[b31] Laass MW, Hennies HC, Preis S, Stevens HP, Jung M, Leigh IM (1997). Localisation of a gene for Papillon-Lefèvre syndrome to chromosome 11q14-q21 by homozygosity mapping. Hum. Genet.

[b32] Lefèvre C, Blanchet-Bardon C, Jobard F, Bouadjar B, Stalder JF, Cure S (2001). Novel point mutations, deletions, and polymorphisms in the cathepsin C gene in nine families from Europe and North Africa with Papillon-Lefe˓vre syndrome. J. Invest. Dermatol.

[b33] Lundgren T, Renvert S (2004). Periodontal treatment of patients with Papillon-Lefe˓vre syndrome: a 3-year follow-up. J. Clin. Periodontol.

[b34] Nakajima K, Nakano H, Takiyoshi N, Rokunohe A, Ikenaga S, Aizu T (2008). Papillon-Lefe˓vre syndrome and malignant melanoma: a high incidence of melanoma development in Japanese palmoplantar keratoderma patients. Dermatology.

[b35] Nakano A, Nomura K, Nakano H, Ono Y, LaForgia S, Pulkkinen L (2001). Papillon-Lefevre syndrome: mutations and polymorphisms in the cathepsin C gene. J. Invest. Dermatol.

[b36] Nitta H, Wara-Aswapati N, Lertsirivorakul J, Nakamura T, Yamamoto M, Izumi Y (2005). A novel mutation of the cathepsin C gene in a Thai family with Papillon-Lefevre syndrome. J. Periodontol.

[b37] Noack B, Gorgens H, Hoffmann T, Fanghanel J, Kocher T, Eickholz P (2004). Novel mutations in the cathepsin C gene in patients with prepubertal aggressive periodontitis and Papillon-Lefevre syndrome. J. Dent. Res.

[b38] Noack B, Görgens H, Hempel U, Fanghänel J, Hoffmann T, Ziegler A (2008a). Cathepsin C gene variants in aggressive periodontitis. J. Dent. Res.

[b39] Noack B, Görgens H, Schacher B, Puklo M, Eickholz P, Hoffmann T (2008b). Functional cathepsin C mutations cause different Papillon-Lefèvre syndrome phenotypes. J. Clin. Periodontol.

[b40] Pallos D, Acevedo AC, Mestrinho HD, Cordeiro I, Hart TC (2010). Novel cathepsin C mutation in a Brazilian family with Papillon-Lefèvre syndrome: case report and mutation update. J. Dent. Child. (Chic.).

[b41] Papillon PH, Lefèvre P (1924). Deuxcas de kératodermiepalmaire et plantairesymétriquefamiliale (maladie de Meleda) chez le frère et la soeur. Coexistence dans les deuxcasd'altérations dentaires graves. Bull. Soc. Fr. Dermatol. Vénéorol. Paris.

[b42] Paris A, Strukelj B, Pungercar J, Renko M, Dolenc I, Turk V (1995). Molecular cloning and sequence analysis of human preprocathepsin C. FEBS Lett.

[b43] Pham CT, Ivanovich JL, Raptis SZ, Zehnbauer B, Ley TJ (2004a). Papillon-Lefevre syndrome: correlating the molecular, cellular, and clinical consequences of cathepsin C/dipeptidyl peptidase I deficiency in humans. J. Immunol.

[b44] Piirilä H, Väliaho J, Vihinen M (2006). Immunodeficiency mutation databases (IDbases). Hum. Mutat.

[b45] Pilger U, Hennies HC, Truschnegg A, Aberer E (2003). Late-onset Papillon-Lefe˓vre syndrome without alteration of the cathepsin C gene. J. Am. Acad. Dermatol.

[b46] Rai R, Thiagarajan S, Mohandas S, Natarajan K, ShanmugaSekar C, Ramalingam S (2010). Haim Munk syndrome and Papillon Lefevre syndrome – allelic mutations in cathepsin C with variation in phenotype. Int. J. Dermatol.

[b47] Romero-Quintana JG, Frías-Castro LO, Arámbula-Meraz E, Aguilar-Medina M, Dueñas-Arias JE, Melchor-Soto JD (2013). Identification of novel mutation in cathepsin C gene causing Papillon-Lefèvre Syndrome in Mexican patients. BMC Med. Genet.

[b48] Selvaraju V, Markandaya M, Prasad PV, Sathyan P, Sethuraman G, Srivastava SC (2003). Mutation analysis of the cathepsin C gene in Indian families with Papillon-Lefe˓vre syndrome. BMC Med. Genet.

[b49] Toomes C, James J, Wood AJ, Wu CL, McCormick D, Lench N (1999). Loss-of-function mutations in the cathepsin C gene result in periodontal disease and palmoplantar keratosis. Nat. Genet.

[b50] Turk D, Janjic V, Sitern I, Podobnik M, Lamba D, Dahl SW (2001). Structure of human dipeptidyl peptidase I (cathepsin C): exclusion domain added to an endopeptidase framework creates the machine for activation of granular serine proteases. EMBO J.

[b51] Wani AA, Devkar N, Patole MS, Shouche YS (2006). Description of two new cathepsin C gene mutations in patients with Papillon-Lefevre syndrome. J. Periodontol.

[b52] Wen X, Wang X, Duan X (2012). High immunoglobulin E in a Chinese Papillon-Lefèvre syndrome patient with novel compound mutations of cathepsin C. J. Dermatol.

[b53] Yang Y, Bai X, Liu H, Li L, Cao C, Ge LJ (2007). A novel mutation of cathepsin C gene in two Chinese patients with Papillon-Lefevre syndrome. J. Dent. Res.

[b54] Zhang Y, Lundgren T, Renvert S, Tatakis DN, Firatli E, Uygur C (2001). Evidence of a founder effect for four cathepsin C gene mutations in Papillon-Lefevre syndrome patients. J. Med. Genet.

[b55] Zhang Y, Hart PS, Moretti AJ, Bouwsma OJ, Fisher EM, Dudlicek L (2002). Biochemical and mutational analyses of the cathepsin C gene (CTSC) in three North American families with Papillon-Lefevre syndrome. Hum. Mutat.

